# Evaluation of Training on Coping With Perinatal Loss on Undergraduate Midwifery Students' Competence in Bereavement Care

**DOI:** 10.1155/nrp/6684154

**Published:** 2025-10-29

**Authors:** Jiayi Liang, Xuan Zhou, Xiaojia Sun, Qiaoqiao Shen, Yulin Gao

**Affiliations:** ^1^School of Nursing, Southern Medical University, Guangzhou, Guangdong, China; ^2^Department of Critical Care, The Second Affiliated Hospital of Guangzhou Medical University, Guangzhou, Guangdong, China

**Keywords:** bereavement care, midwifery education, perinatal death, undergraduate midwifery students

## Abstract

**Objective:**

To determine participants' pre- and post-training competence in perinatal bereavement care, along with evaluation of the training.

**Methods:**

A quasi-experimental study with a pre–post design was conducted in two phases. The training on coping with perinatal loss (TCPL) was first delivered entirely online to a cohort of students in 2022(*N* = 56). To enhance the initial intervention, a second cohort of students in 2023(*N* = 44) received the TCPL with an added on-site scenario simulation component. Questionnaires of Perinatal Bereavement Care Competence Scale, Self-Competence in Death Work Scale, Jefferson Scale of Empathy, Chinese version of the Stress-Related Growth Scale-Short Form, and the evaluation of scenario simulation were used to evaluate the effectiveness of the training.

**Results:**

Including 52 junior undergraduate midwifery students in 2022 and 44 in 2023 completed the questionnaires. We observed higher post-training scores for perinatal bereavement care competence and stress-related growth, and a high degree of satisfaction in the scenario of simulations.

**Conclusions:**

The results support the feasibility of the training and are consistent with short-term improvements; confirmatory controlled studies are warranted.

**Trial Registration:**

Chinese Registry of Clinical Trials: ChiCTR2200066048

## 1. Introduction

Perinatal loss, including miscarriage, stillbirth, and neonatal death, occurs between 20 weeks of gestation and 7 days postpartum. According to estimates by UNICEF, the global stillbirth rate in 2021 was 13.91 stillbirths per 1000 total births, and the global neonatal mortality rate was 17.67 death per 1000 live birth. In comparison, China reported 4.90 and 3.21, respectively [1]. Bereaved families often experience short-term or even long-term grief [2]. And the nursing they receive plays a vital role in their grieving recovery process [2–5].

Losses in the second and third trimesters are usually assisted by midwives, often in the delivery room [6]. Previous studies have indicated that midwifery students were often unprepared, and experienced considerable shocked and surprised when they first confronted with a perinatal loss incident in clinical practice. In response, they tended to avoid engaging in in-depth conversations, largely due to concerns that their words or actions might further exacerbate the distress of the bereaved women [2, 6–13]. They may feel unprofessional about their true feelings, resulting in self-blame and emotional suppression [2, 6–13]. The above are typical manifestations of professional grief [14]. The truth, however, is that these responses are often underappreciated, and coupled with some clinical faculty's avoidance or platitudes, midwifery students strive to perform as expected, and disenfranchised grief ensues [11, 15]. These backlogs of negative experiences might undermine their confidence in their day-to-day performance, reduce their professional identity, and leave them at risk of burnout, which could affect their personal career development [8, 11, 12, 14, 16]. Some midwives said this painful experience would never be forgotten [16]. Accordingly, it may be too late to receive education after encountering perinatal loss in the clinical setting, and midwifery students are suggested to be trained as early as possible so that they can be previously prepared to provide grief support to suffered families [16–18].

## 2. Background

As far as we know, researchers have carried out perinatal bereavement care education among midwives or midwifery students [19, 20]. Hollins et al. [21] developed an interactive workbook and found that it improved midwifery students' learning perception of bereavement. Additional research has explored the positive impact on midwifery students' views of perinatal loss through poetry and collaborative art projects [17, 22]. Furthermore, various teaching methods such as scenario simulation, role-playing, debriefing, and workshops were adopted to provide with a safe and clinically realistic learning experience, and positive results also appeared [9, 16]. These studies fully illustrated the feasibility of applying perinatal bereavement care education to midwifery students [20].

In training related to perinatal loss, cultural distinction in the topics of death and grief should be considered. Existing studies focus on Western countries, where much attention has been focused on perinatal loss events, such as the United Kingdom, the United States, Ireland, and Australia [19, 20]. In mainland China, there have not been domestic training reports for undergraduate midwifery students. Therefore, based on Swanson's caring theory, constructivism learning theory, and experiential learning theory [23, 24], we developed training on coping with perinatal loss (TCPL), taking into account the evidence from perinatal bereavement care international guidelines and Chinese cultural characteristics [6, 25, 26].

In terms of evaluation, tools designed based on the specific learning contents are difficult to be applied directly to other educational programs [15]. Meanwhile, cultural differences also limit the clinical application of some items in China. Therefore, this study used the Perinatal Bereavement Care Competence Scale, which was scientifically and rigorously developed based on previous results of qualitative interviews with Chinese midwifery and a systematic review of international guidelines on perinatal loss [27], as the primary outcome measure.

## 3. Methods

### 3.1. Design

A single-group, pre–post-test design was used to examine the TCPL among junior undergraduate midwifery students. This design estimates within-cohort pre–post differences and does not support causal claims. The concrete aims were to evaluate the impact and experience with the TCPL on the primary outcome of perinatal bereavement care competency level, secondary outcomes of self-competence in death work, empathy, and stress-related growth. Under the influence of COVID-19, the scenario simulation part of the TCPL was forced to be completed online in November 2022. To compensate for this deficiency, we repeated the TCPL in another group of similar midwifery students in November 2023 and completed the scenario simulation offline. The 22-item Transparent Reporting of Evaluations with Nonrandomized Designs was used as a guideline to ensure accurate and complete reporting of the study results [28] (see Supporting Information [available here]).

### 3.2. Theoretical Framework

The design and evaluation of the TCPL is grounded in Swanson's caring theory, constructivism learning theory, and experiential learning theory [23, 24]. Swanson's caring theory provided the overarching philosophical foundation for compassionate, family-centered bereavement care [23]. Constructivism learning theory informed the pedagogical approach, emphasizing that learning is an active process of constructing knowledge through interaction and authentic experiences [24]. Experiential learning theory provided the structural process for skill acquisition and reflection, outlining the cyclical learning journey from experience to application [24]. The following subsections detail how these theoretical principles were concretely applied in the intervention's development and implementation.

### 3.3. The TCPL Intervention

#### 3.3.1. Participants

Convenience sampling was used, recruiting all 56 midwifery students in November 2022 and all 44 in November 2023, both in the third year of the Bachelor of Midwifery program at a medical university before initial clinical exposure, except those on leave. This university became one of the first batches of midwifery undergraduate professional training colleges in China in 2017, and its midwifery major was also selected as the first batch of provincial first-class undergraduate major construction sites by the Ministry of Education in 2019. All participants had multiple experiences with low- and high-fidelity simulations throughout their previous study.

#### 3.3.2. Goal

We select junior undergraduate midwifery students to help them prepare psychologically and intellectually before their initial encounter with perinatal loss events, improve their coping skills, and buffer against the impact of perinatal loss events on them. We chose this goal to address issues identified by previous midwifery interns, such as unavoidable anxiety and avoidance response, extreme fear, self-blame, and emotionally suppressed situations [6].

#### 3.3.3. Development

In TCPL, we first proposed professional grief and self-adjustment methods for medical staff and expanded to the professional grief of midwives and midwifery interns in perinatal loss. Subsequently, Swanson's caring theory was operationalized, which was achieved by structuring content around its five process: based on “maintaining beliefs,” fully assessing to “knowing” the needs of perinatal families, sharing emotions through “being with,” and finally implementing care through “doing for” and “enabling” to help midwifery students deeply understand personalized bereavement care [23]. To implement the principles of constructivism learning theory, teaching methods such as case analysis, experience sharing, and situational simulation were adopted to bridge the gap between theory and clinical practice and engage midwifery students' attention [24]. Furthermore, adopting experiential learning theory provides a clear learning cycle: concrete experience, reflective observation, abstract conceptualization, and active experimentation [24]. Midwifery students can better experience the entire learning process through these four parts. With the efforts of the instruction team (including two associate professors, one full-time lecturer, one doctoral student, and three master's students), 3 modules were generated based on the relevance and sequence of learning (Table 1), with 2 theoretical courses and one scenario simulation. The instruction team prepared and agreed uniformly on the debriefing procedure for scenario simulation to ensure the consistency of instruction.

#### 3.3.4. Implementation

TCPL was completed within 3 weeks. Each module is about 100 min. An associate professor with more than 18 years of experience in teaching death education to undergraduate medical students taught Modules 1 and 2. In these 2 modules, theoretical teaching and case studies were adopted. For midwifery students to freely arrange their learning tasks, these 2 modules have been recorded and uploaded to the university's AIKE Teaching Support Platform (https://aike.smu.edu.cn/). AIKE Teaching Support Platform is based on Moodle, the world's top open-source learning management platform, aiming to provide an open, shared, and collaborative online teaching environment for teachers and students in the school.

In Module 3, four subscenario simulations were included, led by an instruction team of three full-time nursing teachers and one master's student assistant (majoring in perinatal bereavement care research). Midwifery students were freely divided into 3 groups, with each teacher leading one group (approximately 14 to 15). Four sequential subsimulations' case information was distributed to the midwifery students in advance for preparation. In the scenario simulation, they first reviewed the learning content of the previous 2 modules, then started the role-playing part, and finally ended with a guided debriefing.

##### 3.3.4.1. Pilot Study

We conducted a pilot study on 15 junior midwifery students before clinical placement in June 2022. The results indicate that their perinatal bereavement care competence and self-competence in death work have been improved. Meanwhile, the training was highly evaluated by all participants, preliminarily determining the feasibility and acceptability of the TCPL among undergraduate midwifery students.

##### 3.3.4.2. November 2022

Influenced by COVID-19, TCPL was modified after consideration by the instruction team. In the scenario simulation section, we replaced the personal performances with watching role-playing videos (recorded during the pilot study with the consent of participants).

##### 3.3.4.3. November 2023

The recruited 44 midwifery students received a combination of online and offline teaching. In addition to the online courses in Modules 1 and 2, the scenario simulation section was conducted on-site, and midwifery students had the opportunity to personally simulate the scenario and observe the behavior of other role-playing actors up close.

### 3.4. Ethics

The Medical Ethics Board at the University approved the study (Ethics Committee of Southern Medical University [2022] No. 23), and all participants provided written consent. All data related to participants were anonymized and kept confidentiality. Due to the sensitive nature of the study, emotional support was put in place for the duration of the training.

### 3.5. Data Collection

At the beginning of TCPL, midwifery students were provided a perinatal loss case, asked to evaluate their stress level if they were the midwife who handled this case, and selected the appropriate responses to the perinatal loss case through Wenjuanxing (an electronic questionnaire platform). In the meantime, baseline data were collected, including demographic characteristics questionnaire, measurement tools of the Perinatal Bereavement Care Competence Scale, Self-Competence in Death Work Scale (SC-DWS), and Jefferson Scale of Empathy (JSE). After the TCPL, participants were asked again about the stress level and responses of the same perinatal loss case, and the outcome data were collected, in addition to the previous scale, adding the Stress-Related Growth Scale-Short Form (SRGS-SF) and the evaluation of the scenario simulation.

#### 3.5.1. Demographic Characteristics Questionnaire

Self-designed demographic characteristics questionnaire included age, gender, personality, religion, death attitude, family talk about death, bereavement experience, and previous training experience.

#### 3.5.2. Evaluation of Responses to the Perinatal Loss Case

According to the literature review, the self-designed evaluation of responses to the perinatal loss case consisted of (1) a 10-point Likert scale to assess the stress level of the midwife who handled the case (1–10, the higher the score, the more stress); (2) a checklist of the feelings of the dead baby, perinatal loss women and their families.

#### 3.5.3. Measurement Tools

The Perinatal Bereavement Care Competence Scale was developed by Shen et al. [27] to evaluate the ability of midwives' perinatal bereavement care. It consists of 25 items and 6 dimensions: maintaining belief, knowing, being with, preserving dignity, enabling, and self-adjustment. Participants were asked to rate their responses according to the extent to which the items were compatible with their actual thoughts when encountering caring for perinatal loss women. Items are evaluated on a 5-point Likert scale ranging from 1 (*completely incompatible*) to 5 (*completely compatible*). The potential range was between 25 and 125. A greater score presents greater competence in perinatal bereavement care. In the present study, Cronbach's alpha coefficients of the scale for the pre- and post-test were 0.925 and 0.954 in November 2022, and 0.840 and 0.918 in November 2023, respectively.

SC-DWS is a 16-item scale developed by Chan et al. [29]. It is recommended to use the whole scale to assess the self-competence of professionals engaged in death-related work. Participants were required to rate their responses to the extent the items were compatible with their current situation, on a scale ranging from 1, meaning *completely incompatible*, to 5, meaning *completely compatible*. The total score range is 16 to 80 points. The higher the score, the greater the self-competence in death work. Cronbach's alpha coefficients of the scale before and after the intervention in the present study were 0.884 and 0.934 in November 2022, and 0.933 and 0.896 in November 2023, respectively.

JSE was developed by Dr. Mohammadreza Hojat et al. [30] in 2001. In this study, we used the compiled version, which showed good measurement results among nursing students in China [31]. It contains 20 items and 3 dimensions: perspective taking, compassionate care, and walking in the patient's shoes. Items are answered on a 7-point Likert scale ranging from 1 (*strongly disagree*) to 7 (*strongly agree*). Half of the items are scored in reverse. Higher scores are associated with greater empathy. The Cronbach's alpha coefficients for the pre- and post-test in the present study were 0.871 and 0.897 in November 2022, and 0.883 and 0.866 in November 2023, respectively.

SRGS-SF was developed by Park et al. [32] and compiled by Li et al. [33], whose validity and reliability have been examined among Chinese nursing students. It contains 15 items and 2 dimensions: interpersonal growth and intrapersonal growth. Items are evaluated on a 3-point Likert scale ranging from 0 (*not at all*) to 2 (*a great deal*). The total score range is 0 to 30 points. The higher the score, the greater the level of growth. Because of its simplicity, it is welcomed to measure perceived positive changes in responses to stressors [33]. In the present study, Cronbach's alpha coefficient of the scale was 0.920 in November 2022 and 0.901 in November 2023.

In terms of evaluation of the scenario simulation, we adopted 3 evaluation tools designed by the National League for Nursing and Laerdal multisite project group [34] and localized by Wang et al. [35], including the Simulation Design Scale (SDS), the Educational Practices in Simulation Scale (EPSS), and the Student Satisfaction and Self-Confidence in Learning Scale (SSS). SDS contains 20 items and 5 dimensions: objectives/information, student support, problem solving, guided reflection and feedback, and fidelity. EPSS consists of 16 items to measure the best practices in the instructor-developed simulation, divided into 4 elements: active learning, diverse ways of learning, high expectations, and collaboration. In addition, SSS is a 13-item instrument, containing 2 dimensions: satisfaction with simulation activity and self-confidence in learning. All 3 instruments use the 5-point Likert scale, from 1 (*strongly disagree*) to 5 (*strongly agree*), and the average dimension score was calculated. The Cronbach's alpha coefficient calculated for SDS, EPSS, and SSS in the present study ranged from 0.847 to 0.967 in November 2022, and 0.880 to 0.941 in November 2023.

### 3.6. Analysis

Data analysis set *α* = 0.05 as the significance level. Descriptive statistics (frequencies, percentages, measures of central tendency, and measures of variability) were used to summarize results from the samples' demographic characteristics and measurement tools used in the study. Pre- and post-test scores were created for each vignette. Paired *t*-test, Wilcoxon signed rank test, and McNemar chi-square test were applied to determine statistically significant differences between paired data. Independent samples *t* tests were performed to compare the differences between online and offline courses. According to intention to treat (ITT), all participants who completed the entire training processes, and filled out at least one preintervention or postintervention questionnaire survey will be included in data analysis. Missing values will be filled in using the mean or median of preintervention or postintervention scores, respectively.

## 4. Results

### 4.1. TCPL 2022 Feedback

All 56 undergraduate midwifery students participated in the TCPL 2022, of which 52 completed baseline measurements and 49 completed post-test measurements. Therefore, the results presented below were based on the responses of 52 midwifery students.  Table 2 shows the detailed demographic characteristics of the participants. Participants were 52 undergraduates (51 women, 1 man), aged 19–22 years; median 20 (IQR20-21). No participants reported a religious affiliation. None reported prior TCPL. Thirty-nine (75.0%) participants had experienced a bereavement, and 13 (25.0%) reported that their families were comfortable discussing death. All of them only learned about death-related topics in basic nursing and nursing ethics, but not systematic death education, nor did they receive education on how to cope with perinatal loss.

Table 3 shows the scores of the Perinatal Bereavement Care Competence Scale, SC-DWS, and JSE before and after the TCPL. Higher post-training scores were observed for midwifery students' perinatal bereavement care competence (Δ = 8.00, 95% CI 4.773 to 11.223, *p* < 0.001), together with the maintaining belief, knowing, being with, preserving dignity, and self-adjustment dimensions (*p* < 0.05). Higher post-training scores were observed for the SC-DWS (Δ = 7.06, 95% CI 4.487 to 9.637, *p* < 0.001). For the dimensions of JSE, perspective taking increased, compassionate care and walking in patients' shoes decreased significantly (*p* < 0.05). The total score of JSE were numerically lower, but the pre–post difference was not statistically significant.

Table 4 shows the comparison of responses to the same perinatal loss case before and after the TCPL. The incidence of feeling overwhelmed and sympathetic toward the perinatal loss woman and feeling sympathetic toward the perinatal loss family increased, while the incidence of feeling angry toward the perinatal loss family was lower post-training (*p* < 0.05).

The median perceived stress level of midwifery students toward perinatal loss events decreased from 7.0 (5.3–8.0) to 6.0 (5.0–7.0), the difference was not statistically significant (*Z* = −1.932, *p*=0.053). As for SRGS-SF, the median scores were 23.5 (19.0–28.0), 13.0 (10.3–15.8), and 10.0 (8.0–13.0) for total stress-related growth, interpersonal growth, and intrapersonal growth, respectively.

Table 5 shows that midwifery students have a median score of 4 or above for each part of the scenario simulation.

### 4.2. TCPL 2023 Feedback

All 44 undergraduate midwifery students participated in the TCPL 2023, of which 35 filled out the pretest and 44 filled out the post-test. Demographics reflect pretest respondents (*n* = 35); post-test outcomes include all post-test respondents (*n = *44). Participants were 35 undergraduates (33 women, 2 men, aged 18–21 years; median 20 (IQR20-20). No participants reported a religious affiliation. Twenty-two (62.9%) participants had experienced a bereavement, and 4 (11.4%) reported that their families were not afraid to talk about death. The training experience related to death education is the same as that of the participants in 2022 (Table 2).

Higher post-training scores were observed for midwifery students' perinatal bereavement care competence (Δ = 5.02, 95% CI 2.275 to 7.771, *p* < 0.001), together with the knowing and preserving dignity dimensions (*p* < 0.05). Higher post-training scores were observed for the JSE (Δ = 10.43, 95% CI 6.143 to 14.715,*p* < 0.001), together with the dimensions of perspective taking and compassionate care (*p* < 0.001). The SC-DWS scores were numerically higher, but the pre–post difference was not statistically significant (Table 3).

The incidence of feeling pity for the perinatal loss baby increased (*p* < 0.05), while the incidences of feeling shocked for the perinatal loss baby and feeling powerless for the perinatal loss woman and the perinatal loss family were lower post-training (*p* < 0.05) (Table 4).

The median perceived stress level of midwifery students toward perinatal loss events decreased from 6.2 (6.0–8.0) to 6.0 (5.0–7.0), but the pre–post difference was not statistically significant (*Z* = −1.362, *p*=0.173). As for SRGS-SF, the median scores were 24.0 (19.3–27.8), 14.0 (11.3–15.0), and 11.0 (8.3, 13.0) for total stress-related growth, interpersonal growth, and intrapersonal growth, respectively.

### 4.3. Comparison of Blend Online and On-Site Learning Feedback

No adverse events occurred among midwifery students in TCPL. There is no statistically significant difference in the general information and perinatal bereavement care competence score before TCPL between the two groups, except for age and personality. After TCPL, there was no statistically significant difference in perinatal bereavement care competence scores between the two groups (*p* > 0.05). In scenario simulation, midwifery students in the online learning group scored higher in satisfaction with simulation activity and self-confidence in learning than those in the on-site learning group (*p* < 0.05). There was no statistical difference in SDS and EPSS (*p* > 0.05).

## 5. Discussion

In this study, most of the midwifery students had experienced bereavement, and only some of their families did not shy away from discussing the topic of death. In addition, a lack of training may make it difficult for them to find appropriate communication and nursing methods from existing knowledge and experience [2]. Therefore, providing relevant learning opportunities can help them prepare for coping and facilitate the smooth implementation of bereavement care services for perinatal loss families in clinical nursing.

Evidence demonstrates that midwifery students gained better competence in perinatal bereavement care through the TCPL, which was commensurate with the findings of the bereavement education workshop [16]. The focus of the TCPL is on grief support for perinatal families. In “Bereavement and its Care Points of Perinatal Loss Women and their Families,” international guidelines for the management of perinatal loss were adopted, and combined with local clinical case analysis, to help midwifery students understand the reasons behind perinatal loss and promote their sense of control over the process of perinatal bereavement care [3, 8]. In “Bereavement Responses in Perinatal Loss Women and their Families,” midwifery students have come to understand and believe that families can cope with the changes brought about by perinatal loss. In “Caring Points for Perinatal Bereavement Care,” midwifery students can focus on the needs and desires of perinatal loss families and provide them with companionship and care. For the personalized needs of families, they can consciously provide necessary information or explanations to help families focus on important issues and improve their self-care abilities [27]. The Perinatal Bereavement Care Competence Scale was used for the first time to examine the effectiveness of perinatal bereavement care training among undergraduate midwifery students. The result revealed that their perinatal bereavement care competence score was at a high level, compared with the median total score of 75.00, and was higher than that of midwives [36], regardless of the pre- and post-TCPL, which was different from previous qualitative studies [6, 7, 18, 20]. In this study, midwifery students only experienced perinatal loss cases through texts and videos of clinical midwives and midwifery interns, lacking real exposure to perinatal loss scenes, which may cause a lower perception of the impact of the perinatal loss, resulting in higher self-assessment.

Meanwhile, SC-DWS was adopted to measure the ability to cope with self-emotional and survival challenges in death work. In TCPL 2022, SC-DWS scores in midwifery students improved, which is consistent with the results of the 3-day workshop for professionals working in death to improve their self-efficacy in death work in Hong Kong [37]. Perinatal loss is often accidental and may not have a specific cause, and the TCPL allows midwifery students to experience the impermanence of life. Since they cannot alleviate the grief caused by perinatal loss, midwifery students should respond with a positive attitude and do their best to provide considerate care for the bereaved families and promote bereavement healing [2, 4, 9, 17]. However, taking care of bereaved parents can often be emotionally challenging for midwifery students [3, 17, 20]. In the TCPL, midwifery students obtained the opportunity to understand midwifery interns' real sentiments, which helped to speculate on their possible reactions and needs when encountering a perinatal loss event. When faced with such an emotionally challenging and death-shock event as perinatal loss events [2, 11, 19], the use of SC-DWS to measure the ability to handle such a situation is in line with the outreach vision of the authors of the SC-DWS development [29]. However, in TCPL 2023, there was no statistically significant improvement in the SC-DWS score, which may be related to the limited sample size.

When facing the perinatal loss case for the first time, midwifery students felt sympathetic, regretful, powerless, grieving, and overwhelmed, which was consistent with the previous research [6]. It is worth noting that in TCPL 2022, the incidence of midwifery students feeling angry toward the bereaved family member has decreased, and the incidence of feeling overwhelmed toward the bereaved woman has increased. The reason may be that, in association with the fleeting nature of fetal life and the sadness of the perinatal loss woman, midwifery students inevitably blame the family members around them for the negligence. However, after the TCPL, they learned about grief support and realized that any perinatal loss, regardless of the cause, would have an impact on the entire family [18]. As a result, their anger toward the family member subsided. During the TCPL, midwifery students have learned that grief reactions are highly personalized, and although they have undergone simulated training, they still feel uncertain about the possible reactions of bereaved women in real situations [9, 11]. And there are still many difficulties in translating acquired knowledge into clinical practice [38], which makes more midwifery students feel overwhelmed.

According to the division of JSE score, midwifery students' pre- and post-TCPL scores were at a high level, which may benefit from the integration of ideological and political education, role-playing, and scenario simulation teaching in various courses [39]. In TCPL 2023, midwifery students' empathy score had improved, indicating that they can fully respect the decisions of perinatal loss families and provide corresponding emotional support. In TCPL 2022, there was no statistically significant change in the empathy score; however, dimensions of compassionate care and walking in patients' shoes decreased significantly. It is a phenomenon of widespread concern in medical education [40–42]. Two consistent explanations have emerged in the literature: one is the inner psychological changes (such as defense mechanisms and coping skills) that individuals produce to protect themselves when adapting to a new environment [41]; the other is that empathy has to be sacrificed to maintain objectivity and accomplish essential tasks. Firstly, due to the emotional challenges of perinatal loss, midwifery students may have learned to protect themselves through emotional disengagement during the self-adjustment part of the training. Thus, the dimension score of walking in patients' shoes declined, which is consistent with the first point. Secondly, perinatal loss women also need regular delivery care. However, due to a lack of clinical experience, they already have uncertainty and nervousness about clinical practice [8, 12, 43]. It is challenging to release the “headspace” required to be aware of the perinatal loss women, thereby reducing the dimension score of compassionate care [44]. Therefore, educators should pay attention to the changes in midwifery students' empathy in teaching activities and provide timely positive guidance.

In this study, the Chinese version of SRGS-SF was first applied to assess the psychological growth of midwifery students after experiencing the perinatal loss events in TCPL. Stress-related growth refers to positive changes caused by stress events [45]. The result reflected remarkable growth, for the score of SRGS-SF was at a high level compared to the median overall score of 15.00, demonstrating the educational effectiveness of professional education. The subjective experience of events is suggested to have an impact on stress-related growth [46]. Stress perception of the perinatal loss case remained at a moderate or above level before and after TCPL in both groups (*p* > 0.05), which indicated that midwifery students perceive positive stress during the learning of perinatal loss [45, 47]. Although the exposure to perinatal loss in TCPL brought new emotional and task challenges, midwifery students have gained some growth by acquiring knowledge and skills from the new training.

As for scenario simulation, midwifery students in TCPL 2022 and TCPL 2023 made a high appraisal of learning satisfaction and rated the simulation teaching design and implementation process highly (Table 5). Further analysis revealed that midwifery students in TCPL 2022 had higher evaluations of satisfaction and self-confidence in learning than those in TCPL 2023. Possible reasons are as follows: on-site scenario simulation for perinatal bereavement care may induce psychological burden because of individual differences. They may be worried about being frustrated again in the discussion and adopt a self-protection and avoidance attitude, failing to fully engage in teaching activities. Previous studies have found that stigmatization related to mental health issues may become a barrier to psychotherapy and positive outcomes, in which patients often refuse psychological counseling due to fear of stigmatization [48]. Therefore, for the topic of perinatal loss, online training may bring out more educational benefits than face-to-face teaching. Online training can effectively reduce the psychological burden caused by face-to-face teaching and help individuals encounter and solve their problems truthfully [48]. However, the inevitable lack of teacher–student interaction and low student participation in online teaching should be taken seriously. The researchers suggest that in the future, the scenario simulation can continue to adopt the alternative method in this study, and be performed by standardized patients to increase the realism of the situation and improve midwifery students' experience [49].

### 5.1. Implications

This study presents the first structured training program in mainland China designed to prepare undergraduate midwifery students for perinatal loss events. Its educational significance lies in its preventive and protective approach: implementing TCPL before initial clinical exposure equips midwifery students with essential competencies and resilience, thereby reducing the potential negative psychological impact of encountering perinatal loss events in practice.

During the COVID-19 pandemic, strict lockdown measures necessitated that TCPL 2022 be conducted entirely online. However, by 2023, we successfully resumed on-site simulations, effectively overcoming previous limitations. This study indicates that although the pandemic presented operational challenges, it also accelerated the adoption and validation of diverse instructional models, suggesting feasibility across delivery modalities in this context. It is noteworthy that compared with TCPL 2022 (online), TCPL 2023 (blended) showed larger observed pre–post differences on some measures; these between-cohort comparisons are exploratory and may reflect delivery-mode and cohort differences.

Furthermore, the observed pre-post improvements associated with TCPL within a nationally accredited, standardized undergraduate program in midwifery highlight its strong potential for scalability and integration. When considering implementation, institutions should prioritize facilitators' expertise in death education, ensure adequate simulation resources, and cultivate sensitivity toward students' norms about discussing death at home to create a supportive and psychologically safe training setting. Participants' systematic baseline education suggests that this training is not only feasible but also highly generalizable to other midwifery programs across mainland China. Therefore, we recommend that perinatal bereavement care training, modeled on the TCPL framework, be incorporated into the core national midwifery curriculum. This would ensure that all future midwives enter clinical practice with foundational preparedness to provide compassionate and competent care in perinatal loss events, ultimately improving the quality of care for bereaved families and the well-being of healthcare providers alike.

### 5.2. Strengths and Limitations

A key strength of this study lies in the rigorous, iterative development and evaluation of the TCPL. The intervention was designed through a robust synthesis of Swanson's caring theory, established educational principles, international guidelines, and crucial cultural adaptations for the Chinese context. This development process was further advanced through a two-phase study design, allowing for the enhancement of the initial program with an on-site scenario simulation to improve its evaluation. Furthermore, the use of a theory-derived and culturally adapted scale, developed through a rigorous process involving qualitative studies and systematic reviews, ensured that the primary outcome was measured with high reliability and validity, enhancing the credibility of the training effects.

This study has several limitations. Given the single-group pre–post design without a control, causal inference is limited; observed changes may reflect secular trends, maturation, or testing effects. Between-cohort (2022 vs. 2023) contrasts are exploratory and potentially confounded by delivery mode and cohort characteristics. Furthermore, the sample size was small due to constraints in sample sources and geographical coverage. A notable limitation is the significant gender imbalance within our sample; for example, the TCPL 2022 cohort consisted of 51 women participants and only one man. This distribution, while representative of the current student population in midwifery, limits the generalizability of our findings to male students. Future studies should incorporate a comparison group, randomization, and follow-up.

## 6. Conclusion

Participation in the TCPL was associated with higher post-training scores on perinatal bereavement care competence. These preliminary findings suggest that the program has the potential to better prepare students to understand the impact of perinatal loss and to provide supportive care. Further controlled studies with controlled studies with long-term follow-up are needed.

## Figures and Tables

**Table 1:** Modules and training objectives of the TCPL.

Module	Training objectives
Module 1: Professional grief of medical staff and its adjustment • Theoretical knowledge about grief • Professional grief in medical staff • Midwifery interns' feelings about facing perinatal loss events • Self-adjustment of professional grief	To help midwifery students understand professional grief and cope psychologically with provided effective support. 1. Recognition: i. Definition of loss, bereavement, grief, mourning ii. Common manifestation of the normal and complex grief. 2. Comprehension: i. Theory of grief response. ii. Difference between normal grief and complex grief. 3. Application: i. How to cope with others' grief response. ii. How to self-adjust for professional grief for medical staff.

Module 2: Bereavement and its care points of perinatal loss women and their families • The specificity of perinatal loss • Bereavement responses in perinatal loss women • Bereavement responses in perinatal loss family • Principles of perinatal bereavement care • Caring points for perinatal bereavement care	To help midwifery students understand the bereavement response of perinatal loss families and master the bereavement caring points 1. Recognition: i. Principles of bereavement care for perinatal loss families. 2. Comprehension: i. Common bereavement responses in perinatal loss women and their family members. ii. Bereavement care points for perinatal loss women. 3. Application i. Identify grief responses in women with perinatal loss. ii. Provide individual bereavement care for women with perinatal loss.

Module 3: Scenario simulation • Scenario simulation: Bereavement care for a perinatal bereaved family with induced labor due to fetal diagnosis of diaphragmatic hernia	To enable midwifery students to practice bereavement caring for perinatal loss. 1. Application i. Correctly identify and effectively deal with the bereavement of perinatal bereaved mothers and their families. ii. Effectively develop and use memory lists. iii. Adopt appropriate nonpharmacological labor analgesia for perinatal loss women. iv. Correctly carry out lactation suppression, postpartum examination, and health care guidance for re-pregnancy.

**Table 2:** Demographic characteristics of the participants.

Characteristic		*n* (%)	
		TCPL 2022 (*n* = 52)	TCPL 2023 (*n* = 35)
Personality	Very pessimism	0 (0.0)	0 (0.0)
	Comparative pessimism	9 (17.3)	5 (14.3)
	Intermediate	19 (36.5)	6 (17.1)
	Comparative optimism	21 (40.4)	22 (62.9)
	Very optimism	3 (5.8)	2 (5.7)

Death attitude	Very avoidance	0 (0.0)	0 (0.0)
	Comparative avoidance	14 (26.9)	10 (28.6)
	Intermediate	16 (30.8)	12 (34.3)
	Comparative acceptance	18 (34.6)	11 (31.4)
	Very acceptance	4 (7.7)	2 (5.7)

Talking about death at home	Never	2 (3.9)	2 (5.7)
	Try to avoid	24 (46.2)	18 (51.4)
	Discomfort when talking	13 (25.0)	11 (31.4)
	Pretty open	13 (25.0)	4 (11.4)

Bereavement experience	Yes	39 (75.0)	22 (62.9)
	No	13 (25.0)	13 (37.1)

Perinatal loss coping training experience	Yes	0 (0.0)	0 (0.0)
	No	52 (100.0)	35 (100.0)

*Note:* Demographics reflect pretest respondents (*n* = 35) in 2023; post-test outcomes include all post-test respondents (*n = *44).

**Table 3:** Scores comparison of measurement tools at pre- and post-TCPL.

Outcome		TCPL2022 (*n* = 52)			TCPL2023 (*n* = 44)		
		Mean (SD)	*t* (95% CI)	*p* value	Mean (SD)	*t* (95% CI)	*p* value
*Perinatal bereavement care competence scale*							
Total score	Before	101.63 (10.21)	4.979 (4.773, 11.223)	**< 0.001**	101.00 (6.10)	3.686 (2.275, 7.771)	**< 0.001**
	After	109.63 (9.77)			106.02 (8.36)		
Maintaining belief	Before	13.00 (1.79)	2.633 (0.160, 1.187)	**0.011**	13.00 (1.08)	1.677 (0.078, 0.851)	0.101
	After	13.67 (1.26)			13.39 (1.33)		
Knowing	Before	15.31 (2.21)	4.625 (0.877, 2.222)	**< 0.001**	15.57 (1.70)	2.69 (0.187, 1.307)	**0.010**
	After	16.86 (1.83)			16.32 (1.57)		
Being with	Before	24.88 (3.19)	3.142 (0.565, 2.564)	**0.003**	25.09 (2.35)	0.304 (−0.799, 1.082)	0.763
	After	26.45 (2.69)			25.23 (2.56)		
Preserving dignity	Before	14.94 (2.01)	8.911 (2.211, 3.497)	**< 0.001**	14.91 (1.30)	7.179 (1.827, 3.254)	**< 0.001**
	After	17.80 (1.67)			17.45 (1.84)		
Enabling	Before	21.63 (2.33)	1.136 (−0.374, 1.350)	0.261	20.77 (1.87)	1.676 (−0.134, 1.455)	0.101
	After	22.12 (2.37)			21.43 (2.13)		
Self-adjustment	Before	11.87 (1.67)	3.385 (0.354, 1.384)	**0.001**	11.66 (1.41)	1.571 (−0.155, 1.250)	0.123
	After	12.73 (1.71)			12.20 (1.66)		

*Self-competence in death work*							
Total score	Before	55.02 (9.10)	5.506 (4.487, 9.637)	**< 0.001**	56.66 (8.02)	1.65 (−0.490, 4.903)	0.106
	After	62.08 (9.59)			58.86 (8.56)		
Existential subscale	Before	33.98 (5.85)	5.246 (2.884, 6.460)	**< 0.001**	35.63 (5.34)	1.927 (−0.078, 3.412)	0.061
	After	38.65 (6.17)			37.30 (5.44)		
Emotional subscale	Before	13.52 (2.81)	5.035 (1.075, 2.500)	**< 0.001**	13.69 (2.25)	0.04 (−0.931, 0.967)	0.968
	After	15.31 (2.83)			13.70 (2.85)		

*Jefferson scale of empathy*							
Total score	Before	113.52 (13.50)	−1.125 (−6.277, 1.769)	0.266	103.46 (12.09)	4.907 (6.143, 14.715)	**< 0.001**
	After	111.27 (16.84)			113.89 (11.06)		
Perspective taking	Before	57.04 (6.31)	2.606 (0.521, 4.015)	**0.012**	52.83 (5.69)	3.599 (1.554, 5.516)	**0.001**
	After	59.31 (6.23)			56.36 (5.01)		
Walking in patients' shoes	Before	16.44 (2.92)	−2.814 (−2.471, −0.413)	**0.007**	15.03 (2.79)	1.732 (−0.174, 2.299)	0.090
	After	15.00 (4.37)			16.09 (3.23)		
Compassionate care	Before	40.04 (6.40)	−2.345 (−5.716, −0.443)	**0.023**	35.60 (6.92)	4.426 (3.174, 8.489)	**< 0.001**
	After	36.96 (10.02)			41.43 (5.53)		

*Note:* Higher scores indicate greater perinatal bereavement care competence and self-competence in death work and higher level of empathy. Pre- and post-training scores, *t-*values, 95% CIs, and *p* values are reported for all pre-post differences. The bold text indicates *p* < 0.05.

**Table 4:** Responses comparison of the perinatal loss case at pre- and post-TCPL.

Response			TCPL2022				TCPL2023			
			After		*χ* ^2^	*p* value	After		*χ* ^2^	*p* value
			Yes	No			Yes	No		
*Response to the dead baby, n (%)*										
Shock	Before	Yes	8 (15.4)	12 (23.1)	0.450	0.503	4 (9.1)	24 (54.5)	19.360	**< 0.001**
		No	8 (15.4)	24 (46.2)			1 (2.3)	15 (34.1)		
Fear	Before	Yes	6 (11.5)	10 (19.2)	1.786	0.180	4 (9.1)	8 (18.2)	0.450	0.503
		No	4 (7.7)	32 (61.5)			10 (22.7)	22 (50.0)		
Grief	Before	Yes	34 (65.4)	4 (7.7)	0.750	0.388	30 (68.2)	7 (15.9)	0.056	0.815
		No	8 (15.4)	6 (11.5)			5 (11.4)	2 (4.5)		
Guilt	Before	Yes	11 (21.2)	8 (15.4)	0.308	0.581	5 (11.4)	9 (20.5)	0.083	0.774
		No	5 (9.6)	28 (53.9)			2 (4.5)	28 (63.6)		
Overwhelm	Before	Yes	22 (42.3)	9 (17.3)	< 0.001	> 0.999	18 (40.9)	16 (36.4)	3.273	0.065
		No	10 (19.2)	11 (21.2)			5 (11.4)	5 (11.4)		
Pity	Before	Yes	24 (46.2)	8 (15.4)	1.565	0.201	32 (72.7)	5 (11.4)	4.762	**0.027**
		No	15 (28.9)	5 (9.6)			7 (15.9)	0 (0.0)		

*Response to the perinatal loss woman, n (%)*										
Sympathy	Before	Yes	43 (82.7)	0 (0.0)	5.143	**0.016**	35 (79.5)	1 (2.3)	0.083	0.774
		No	7 (13.5)	2 (3.9)			6 (13.6)	2 (4.5)		
Grief	Before	Yes	20 (38.5)	6 (11.5)	3.682	0.052	20 (45.5)	11 (25.0)	1.562	0.210
		No	16 (30.8)	10 (19.2)			5 (11.4)	8 (18.2)		
Guilt	Before	Yes	9 (17.3)	4 (7.7)	1.231	0.267	1 (2.3)	3 (6.8)	0.444	0.508
		No	9 (17.3)	30 (57.7)			6 (13.6)	34 (77.3)		
Overwhelm	Before	Yes	10 (19.2)	3 (5.8)	5.062	**0.021**	5 (11.4)	4 (9.1)	1.231	0.267
		No	13 (25.0)	26 (50.0)			9 (20.5)	26 (59.1)		
Powerlessness	Before	Yes	25 (48.1)	14 (26.9)	3.368	0.064	21 (47.7)	15 (34.1)	8.471	**0.002**
		No	5 (9.6)	8 (15.4)			2 (4.5)	6 (13.6)		
Anxiety	Before	Yes	0 (0.0)	2 (3.9)	< 0.001	> 0.999	0 (0.0)	3 (6.8)	< 0.001	> 0.999
		No	2 (3.9)	48 (94.2)			2 (4.5)	39 (88.6)		
Angry	Before	Yes	0 (0.0)	3 (5.8)	1.333	0.250	0 (0.0)	4 (9.1)	2.250	0.125
		No	0 (0.0)	49 (94.2)			0 (0.0)	40 (90.9)		
Pity	Before	Yes	28 (53.9)	6 (11.5)	0.267	0.607	19 (43.2)	9 (20.5)	< 0.001	> 0.999
		No	9 (17.3)	9 (17.3)			8 (18.2)	8 (18.2)		

*Response to the perinatal loss families, n (%)*										
Sympathy	Before	Yes	29 (55.8)	2 (3.9)	11.250	**< 0.001**	25 (56.8)	4 (9.1)	2.400	0.119
		No	18 (34.6)	3 (5.8)			11 (25.0)	4 (9.1)		
Grief	Before	Yes	22 (42.3)	6 (11.5)	2.450	0.115	11 (25)	3 (6.8)	2.769	0.092
		No	14 (26.9)	10 (19.2)			10 (22.7)	20 (45.5)		
Guilt	Before	Yes	6 (11.5)	8 (15.4)	0.450	0.503	0 (0.0)	6 (13.6)	< 0.001	> 0.999
		No	12 (23.1)	26 (50.0)			5 (11.4)	33 (75.0)		
Overwhelm	Before	Yes	10 (19.2)	12 (23.1)	0.190	0.664	5 (11.4)	7 (15.9)	0.500	0.481
		No	9 (17.3)	21 (40.4)			11 (25.0)	21 (47.7)		
Powerlessness	Before	Yes	17 (30.9)	15 (27.3)	1.565	0.210	12 (27.3)	17 (38.6)	5.500	**0.017**
		No	8 (15.4)	15 (27.3)			5 (11.4)	10 (22.7)		
Anxiety	Before	Yes	0 (0.0)	5 (9.1)	< 0.001	> 0.999	0 (0.0)	1 (2.3)	3.125	0.070
		No	4 (7.3)	46 (83.6)			7 (15.9)	36 (81.8)		
Angry	Before	Yes	0 (0.0)	7 (12.7)	5.143	**0.016**	0 (0.0)	5 (11.4)	3.200	0.063
		No	0 (0.0)	48 (87.3)			0 (0.0)	39 (88.6)		
Pity	Before	Yes	15 (28.9)	16 (30.8)	2.042	0.152	7 (15.9)	7 (15.9)	1.714	0.189
		No	8 (15.4)	13 (25.0)			14 (31.8)	16 (36.4)		

*Note:* Incidence rates of responses, *x*^2^ values, and *p* values are reported for all pre–post differences. The bold text indicates *p* < 0.05.

**Table 5:** Evaluation of scenario simulation, median (*P*_25_, *P*_75_).

Outcome		Evaluation (score 1–5)		*t* (95% *CI*)	*p* value
		TCPL 2022	TCPL 2023		
SSS	Student satisfaction with the simulation activity	4.20 (4.00, 4.55)	4.00 (3.85, 4.40)	−2.191 (−0.353, −0.017)	**0.031**
	Self-confidence in learning	4.13 (4.00, 4.38)	4.00 (3.75, 4.25)	−2.260 (−0.351, −0.023)	**0.026**

EPSS	Active learning	4.50 (4.03, 4.98)	4.35 (4.00, 4.70)	−0.417 (−0.332, 0.217)	0.678
	Collaboration	4.50 (4.00, 5.00)	4.50 (4.00, 5.00)	0.294 (−0.267, 0.359)	0.770
	Diverse ways of learning	4.50 (4.00, 5.00)	4.00 (4.00, 4.88)	−0.745 (−0.436, 0.198)	0.458
	High expectations	5.00 (4.00, 5.00)	4.50 (4.00, 5.00)	−0.545 (−0.378, 0.215)	0.587

SDS	Objectives/information	4.60 (4.00, 4.95)	4.10 (4.00, 4.60)	−1.084 (−0.410, 0.120)	0.281
	Student support	4.50 (4.00, 5.00)	4.50 (4.00, 4.94)	0.550 (−0.210, 0.371)	0.584
	Problem solving	4.40 (4.00, 4.80)	4.10 (4.00, 4.60)	−0.152 (−0.294, 0.252)	0.879
	Guided reflection and feedback	4.50 (4.00, 5.00)	4.50 (4.00, 4.75)	0.645 (−0.199, 0.390)	0.521
	Fidelity	4.50 (4.00, 5.00)	4.00 (4.00, 4.50)	0.109 (−0.317, 0.354)	0.914

*Note:* Higher scores indicate more favorable evaluations. Statistical comparisons of scores across cohorts (2022 vs. 2023) are presented with *t-*values, 95% CIs, and *p* values. Bold text indicates *p* < 0.05.

Abbreviations: EPSS, Educational Practices in Simulation Scale; SDS, Simulation Design Scale; SSS, Students' satisfaction and self-confidence in learning.

## Data Availability

Data are available upon request.

## Supporting Information

Supporting Information provides the recommendations of the 22-item Transparent Reporting of Evaluations with Nonrandomized Designs guideline followed in this report.
